# Tissue Adhesive versus Skin Suture plus Waterproof Wound Dressings for Carpal Tunnel Wound Closure: A Prospective Randomized Controlled Trial

**DOI:** 10.5704/MOJ.2407.009

**Published:** 2024-07

**Authors:** T Maneesrisajja, K Srikulawong

**Affiliations:** Department of Orthopaedics, Nongkhai Hospital, Nong Khai, Thailand

**Keywords:** carpal tunnel decompression, skin closure, tissue adhesive glue, skin suture, clinical outcomes

## Abstract

**Introduction:**

The popular wound closure methods for carpal tunnel decompression (CTD) include non-absorbable and absorbable sutures which have comparable results in clinical outcomes. However, these wound closure methods are recommended to keep a wound dry which may limit some ADLs. We conducted a prospective randomized controlled trial that compares clinical outcomes and cost-effectiveness in a skin closure following CTD between absorbable sutures plus a 2-octyl cyanoacrylate tissue adhesive (2OCA) versus non-absorbable skin sutures plus a waterproof dressing (NSPWD).

**Materials and Methods:**

We enrolled 120 patients undergoing CTD into two groups: 2OCA and NSPWD, with 60 patients in each group. Number of dressing changes, Quick DASH, pain VAS, cosmetic VAS, patient satisfaction VAS, and Hollander wound evaluation score, cost-effectiveness, and post-operative complications were collected at pre-operative period and two and six weeks post-operatively.

**Results:**

Slightly better patient satisfaction VAS (7.9 vs 7.2, p=0.018) and cosmetic VAS (8.0 vs 7.2, p=0.025) were observed in 2OCA at 2 weeks. Meanwhile, NSPWD revealed lesser times of dressing change (Median, mode, IQR: 0/0/0 vs 2/3/2, p<0.001). The total wound-related costs include dressing change and suture removal cost ($15.9 for 2OCA vs $19.2 for NSPWD, p=0.002) although an initial wound-related cost in 2OCA was higher ($15.7/case vs $7.9/case, p<0.001).

**Conclusion:**

Our study revealed that the supplementary tissue adhesive to absorbable sutures following CTD could reduce total wound-related costs while clinical outcomes might not be considered clinically significant.

## Introduction

Carpal tunnel syndrome (CTS) is the most common compressive neuropathy^1,2^. It can cause functional disability and morbidity. The median number of sick leave days for CTS is among the highest at 27 days^3^. Carpal tunnel decompression (CTD) improved clinical symptoms in 60%–95% of patients^4,5^.

The most commonly employed wound closure methods after CTD include absorbable and non-absorbable sutures. Non-absorbable sutures are a traditional closure method and may be less likely to evoke an inflammatory response or premature separation. Meanwhile, absorbable sutures have become increasingly popular, do not require removal, and may, therefore, save outpatient department (OPD) visiting time and reduce patient anxiety post-operatively^6^.

In recent times, tissue adhesives were introduced and applied in orthopaedic skin closure^7-10^. It acts as a liquid and can rapidly polymerize when contacting with the skin. The tissue adhesive produces a protective layer which may provide advantages, including wound protection from the external environment, antimicrobial protection, the potential for easier self-care (e.g., no need for bandage changes, ability to immediate waterproofing), and no need for OPD visits for suture removal^11-14^.

Previous studies revealed tissue adhesives as an ideal supplement to wound closure following CTD which is easy to use, improves patient satisfaction, and is cost-effective.

At present, only one published study reported on the comparative outcomes of tissue adhesive [n-butyl 2-cyanoacrylate, Indermil®;] and standard skin closure techniques in hand surgery^15^. To our best knowledge, no published studies directly reported on the utilization of 2-octyl cyanoacrylate tissue adhesive (2OCA) as a supplement to wound closure in CTD.

This study aimed to elucidate the role of 2OCA tissue adhesive for supplement skin closure in CTD. We conducted a prospective study to compare the economic, satisfaction, cosmetic, and clinical outcomes of carpal tunnel surgeries with waterproof skin closure through 2OCA tissue adhesive versus skin sutures in CTD.

## Materials and Methods

We performed a prospective comparative study of patients underwent a primary CTD from the elective hand surgery outpatient clinic from December 2021 to May 2022 at the department of orthopaedic. This study was approved by the local ethics committee.

This study included 120 patients. The inclusion criteria were adult patients with primary CTS. Patients who had uncontrolled medical conditions or skin diseases that affect wound healing, other previous palmar surgeries, history of hypertrophic scarring or keloid, known allergy to suture materials, or concurrent immunosuppressive agent treatment were excluded from this study.

**Fig. 1:**
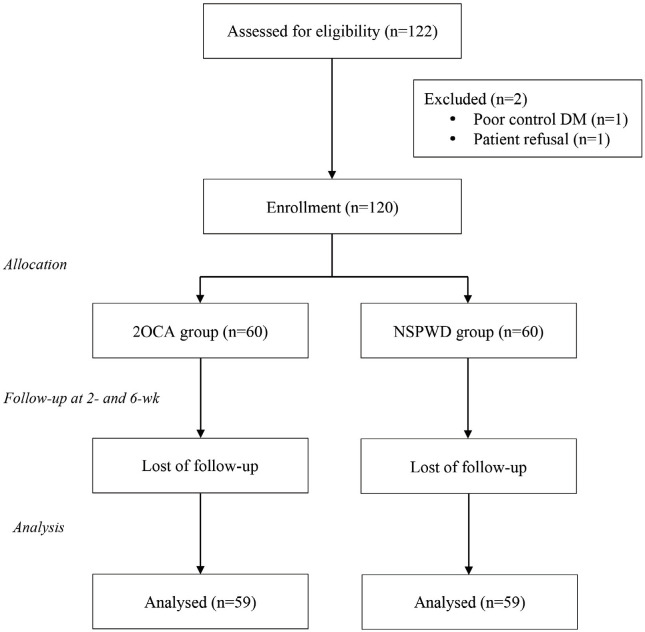
Study flow diagram.

All patients were informed by a research coordinator and obtained consent from the patient. Randomization was performed using a computer-generated block randomization using block sizes of two. The sealed envelopes containing the type of wound closure that would be opened just before closing the wound. The skin was closed using absorbable sutures plus topical 2OCA in the first group whereas the skin was closed using non-absorbable sutures plus waterproof dressing (NSPWD) in the second group (Fig. 1).

**Fig. 2:**
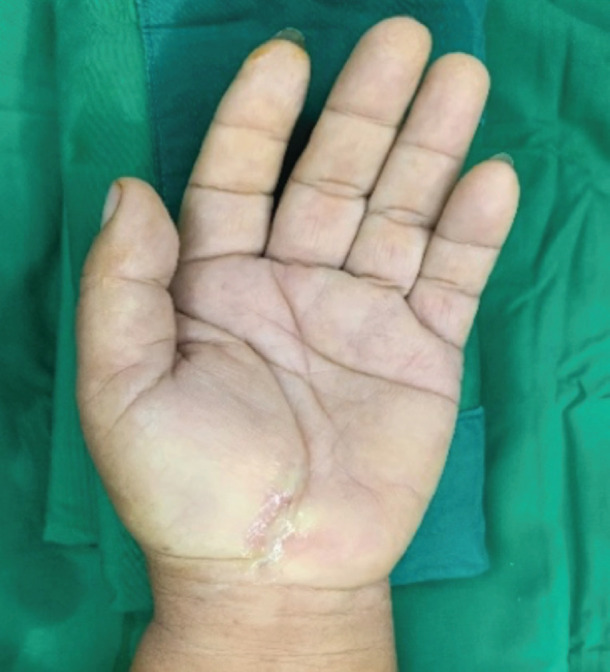
Carpal tunnel wound following closure with absorbable suture plus topical tissue adhesive.

**Fig. 3:**
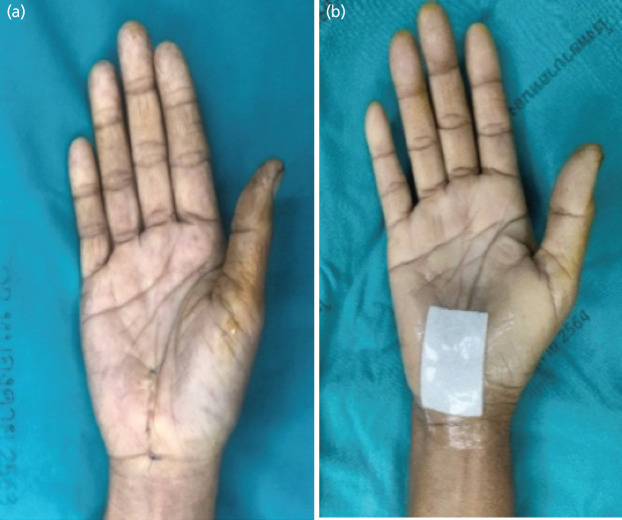
Carpal tunnel wound following closure with nonabsorbable suture plus waterproof occlusive dressing.

All procedures were performed under local anaesthesia using 2% plain lidocaine solution infiltrated into the incision line. A tourniquet was used in all patients. All procedures were performed by either of the two authors (TM and KS). An incision was made from the distal wrist flexor crease in line with the radial border of the ring finger, approximately 3.5cm in length up to Kaplan’s cardinal line level. Wound closure was performed with a continuous subcuticular technique using 4-0 polyglactin 910 [Vicryl Repide®, Ethicon, USA], then a topical tissue adhesive [Dermabond Mini®;, Ethicon, USA] was applied in 2 layers for the 2OCA group (Fig. 2). The wound was closed with 4-0 monofilament polypropylene [Prolene®, Ethicon, USA] plus waterproof occlusive dressing in the NSPWD group (Fig. 3). Patients using non-absorbable suture (NSPWD group) were additional required to use of waterproof occlusive dressings, which are used similarly to the 2OCA group in terms of providing a waterproof barrier over an incision. Compression bandages were removed one day post-operatively, and patients were allowed to do light activities of daily living (ADLs), including taking a shower. Sutures and dressing material were removed at 10 days post-operatively in the NSPWD group.

Patients were pre-operatively assessed with Quick DASH and pain visual analogue scale (VAS). The research coordinator assessed each subject pre-operatively and two and six weeks post-operatively, including the number of dressing changes, Quick DASH, pain VAS, cosmetic VAS, patient satisfaction VAS, and Hollander wound evaluation score (HWES).

All the statistical analyses were performed using Statistical Package for the Social Sciences version 26.0 [SPSS, Chicago, Illinois, USA], and data between 2OCA and NSPWD groups were compared using independent student’s t-test and chi-squared test. Correlations between the cosmetic outcomes and patient satisfaction were evaluated using Spearman’s correlation coefficient. A p-value of 0.05 or less was considered statistically significant.

## Results

This study enrolled 120 patients. Table I shows patient demographic and clinical characteristic data. One patient in each group failed to complete the scheduled follow-up. The remaining 118 patients completed the study with data available for analysis both at 2- and 6-weeks follow-up time points (Fig. 1). All wounds had healed without wound dehiscence or major complication. The mean age of patients in 50.2 and 51.1 years and 67.9% and 58.6% were females in the 2OCA and NSPWD groups, with pre-operative mean DASH scores of 58.7 and 54.3, respectively.

**Table I:** Demographic data compared between the 2OCA and the NSPWD groups.

	2OCA (n=59)	NSPWD (n=59)	p-value
Age (years)	50.2	51.1	0.324
Female: Male	19:10	17:12	0.114
Hand Dominance	16 (55.2%)	15 (51.7%)	0.198

Table II shows the perioperative data of patients in both groups. The mean incision length in the 2OCA and NSPWD groups was 3.6 and 3.4cm (p=0.187), respectively. The mean operative time was significantly longer in the 2OCA group (9.9 min for the 2OCA group vs 8.2 min for the NSPWD group; p<0.001). The times of dressing changes in the NSPWD group were significantly more than that in the 2OCA group (p=0.000) (Table II).

**Table II:** Perioperative data compared between the 2OCA and groups.

	2OCA (n=59)	NSPWD (n=59)	p-value
Incision length (cm)	3.6	3.4	0.187
Operative time (min)	9.9	8.1	0.001
Dressing change (Median, mode, IQR)	0/0/0	2/3/2	0.000
Wound-related complications	1	0	0.322
Initial wound-related cost (Mean±SD) (US dollar)	15.7±1.0	7.9±1.2	<0.001
Total wound-related cost (Mean±SD) (US dollar)	15.9±1.0	19.2±5.6	0.002

During the post-operative period, the times of dressing change in the NSPWD group were significantly more than in the 2OCA group (p=0.000) (Table II). Wound-related cost in the NSPWD group was significantly higher than in the 2OCA group (p=0.002). This cost is related to the time of dressing changes. Wound-related cost excluding dressing change cost was less in the NSPWD group than in the 2OCA group ($7.9/case for NSPWD vs $15.7/case for 2OCA, p<0.001).

Table III shows no difference in cosmetic result between groups as determined by HWES at 2- and 6-week postoperative (p=0.123 and p=0.758, respectively). Meanwhile, the mean cosmetic VAS of the healed surgical incision was significantly greater in the 2OCA group at 2 weeks (p=0.025) but no different at 6-week follow-ups (p=0.478).

**Table III:** Outcome measurement compared between the 2OCA and NSPWD groups.

	2OCA (n = 59)	NSPWD (n = 59)	p-value	2OCA (n=59)	NSPWD (n=59)	p-value
HWES	1.2±0.7	1.5±;0.9	0.123	0.21±0.4	0.24±0.4	0.758
Cosmetic VAS	8.0±1.3	7.2±1.3	0.025	8.9±1.0	8.7±1.2	0.478
Patient satisfaction VAS	7.9±1.2	7.2±0.9	0.018	8.7±0.9	8.4±1.0	0.323
Pain VAS	3.8±1.5	3.6±1.6	0.618	1.3±0.9	1.5±0.8	0.544
Quick DASH score	26.4±12.6	28.0±14.6	0.674	9.7±6.8	10.7±7.6	0.612

The mean perioperative pain VAS was not significantly different between the two groups operatively and post-operatively (p=0.186 at pre-operative period, p=0.618 at 2 weeks, p=0.544 at 6 weeks follow-ups). No difference was found in functional outcomes between groups as determined by QuickDASH score at the operative period and 2 and 6 weeks post-operatively (p=0.364 at the pre-operative period, p=0.674 at 2 weeks, p=0.612 at 6 weeks follow-ups). However, the mean patient satisfaction VAS was significantly better in the 2OCA group at 2 weeks (p=0.018) but no difference at 6 weeks follow-ups (p=0.323).

The evaluation of the relationship between patient satisfaction VAS and cosmetic outcomes revealed a significant association between patient satisfaction VAS and HWES. Conversely, a significant correlation was found between satisfaction VAS and cosmetic VAS only at 2 weeks (r=0.363, p=0.05) but no significant association at 6 weeks follow-up (r=0.202, p=0.128) (Table IV).

**Table IV:** Patient satisfaction as calculated by cosmetic VAS and HWES between 2- and 6-weeks post-operative follow-ups.

	Patient satisfaction VAS			
	2 weeks follow-up		6 weeks follow-up	
	r value	p-value	r value	p-value
Cosmetic VAS	0.363	0.005	0.220	0.098
HWES	0.210	0.113	0.202	0.128

A p-value of <0.05 is considered statistically significant, HWES Hollander Wound Evaluation Scale, VAS visual analog scale

One patient from the 2OCA group had superficial wound infections at two weeks which were successfully treated with oral antibiotics. No other complications were recorded.

## Discussion

An ideal method of wound closure would be providing sufficient strength; less wound complication, thereby saving time for OPD visits for wound care; rapid return to normal ADL; and a good cosmetic outcome. Traditionally, wound closure in CTD using non-absorbable sutures results in less post-operative wound inflammation. However, this method causes pain on suture removal and leaves suture marks^16-18^. These factors can result in a less satisfactory cosmetic result being perceived by patients. Recently, a subcutaneous closure with absorbable suture has gained popularity, but inflammatory reactions and scar wounds may be its disadvantages^19,20^. Several studies revealed no significant difference between both absorbable and non-absorbable suture groups for wound satisfaction, aesthetics outcomes, and functional outcomes^17,21,22^.

In the 1980s, a tissue adhesive composed of n-butyl 2-cyanoacrylate was introduced for topical wound closure in children^23^. Recently, a 2-octyl cyanoacrylate is one of the most commonly used, commercially available wound adhesives. It was approved as an alternative to skin wound closure with sutures, staples, or adhesive strips by the United States Food and Drug Administration (FDA) in 1998. Current indications include all simple approximated wounds from surgical incisions or properly cleaned lacerations from trauma and small wound tension. Additionally, it was approved for use in combination with subcuticular sutures for deep and high-tension areas. A tissue adhesive causes fewer inflammatory reactions than sutures, reduces wound infection rates with its antimicrobial properties, and briefly withstands wetness, such as showering and washing, when applied properly^24^. Furthermore, OPD visits are unnecessary for suture removal or bandage changing. These factors make tissue adhesives a cost-effective method for wound repair and improve patient satisfaction^25^.

Only one published study reported on the comparative wound outcomes of a tissue adhesive [n-butyl 2-cyanoacrylate tissue adhesive, Indermil®, Loctite, Ireland] for skin closure and non-absorbable skin suture in hand and wrist surgery. Their study revealed similar wound outcomes without evidence of significant wound infection. However, the result of the study population was not a specific diagnosis of hand and wrist disease. Additionally, three cases of minor wound dehiscence in a tissue adhesive group were reported^15^. FDA recommends a deep and higher tension wound to require subcutaneous sutures.

In our hospital, the patients who underwent CTD were mainly working age and had the high expectation on cosmetic results and rapid recovery for returning to work. We applied the idea of ERAS to promote wound healing, improve patient satisfaction, and rapid ADL recovery. Wound closure is one of the key factors. We preferred using a subcuticular suture, rather than skin interrupted suture because of the cosmetic outcome. However, this standard wound closure requiring wound dressing limits some ADLs, such as showering and hand washing, which feel discomfort from moisture, especially in tropical countries, and take much time for OPD visits for dressing change. Thus, this study was conducted using tissue adhesive as the supplement to subcuticular sutures for skin closure in a waterproof and bandage-free fashion. Furthermore, our method could avoid wound dehiscence using a tissue adhesive alone compared with a previous study.

To our best knowledge, this is the first study to compare patient satisfaction and economic and clinical outcomes between a tissue adhesive (2-octyl cyanoacrylate) as the supplement to subcuticular suture and NSPWD among patients undergoing elective outpatient CTD.

This study revealed a 2–3 times higher number of dressing changes in the NSPWD group than in the tissue adhesive group. The tissue adhesive group has better 2-week patient-reported VAS scores of satisfactions and cosmetic outcomes than those without tissue adhesive. Additionally, the tissue adhesive group required no suture removal, thereby reducing healthcare providers’ workload and patient inconvenience. This might explain why a patient in the tissue adhesive group had a higher satisfaction score than standard wound closure in early post-operative. The initial cost of the 2OCA group is higher than the NSPWD group ($7.9/case for NSPWD vs $15.7/case for 2OCA) but the total cost, including the cost of dressing changes ($5.3/visit) is higher in the NSPWD group. It would take less than 2 times for dressing changes that overtake the total cost of 2OCA.

In the present study has shown statistically significant better in-patient satisfaction and cosmetic VAS in 2OCA group only at two weeks while these parameters were indifference at six weeks. We could not conclude that there was clinically significance difference between 2OCA and NSPWD groups in term of patient satisfaction and cosmetic VAS.

Several studies compared outcomes of absorbable and non-absorbable sutures for wound closure following CTD. No significant difference was found in pillar pain. The study compared the use of absorbable and non-absorbable sutures for open CTD wound closure which had no significant difference in scar tenderness and pillar pain at 6- or 12 weeks^21^. Problems associated with the use of absorbable sutures include a higher rate of wound inflammation. Many studies revealed this method to increase the incidence of extended wound inflammation and stitch suture micro-abscesses, but without a statistical significance compared with non-absorbable sutures at six weeks^18,20,21^. However, other studies revealed a decreased incidence of wound inflammation reactions and stitch abscesses with the use of Polyglactin910 [Vicryl Rapide®]^6^. Additionally, our study suggests no difference in residual wound pain and inflammatory reaction between the two types of sutures following open carpal tunnel release.

This study has several limitations. To begin with, this study only investigated outcomes in post-operative six weeks. The short follow-up periods were insufficient for a more detailed analysis. However, the results of both groups in the six-week follow-up were not different which may necessitate further follow-up and it would be inconvenient for patients to return visit several months later. Furthermore, we could not conduct this study with a head-to-head comparison between a standalone tissue adhesive and a skin suture because the FDA recommends using a tissue adhesive in combination with subcuticular sutures for deep and high-tension areas. Lastly, the medical material costing method in our hospital might be different from others, so the cost-performance of dressing changes and tissue adhesive would vary among other hospitals.

## Conclusion

Our study revealed that the supplementary tissue adhesive to absorbable sutures following CTD could reduce total wound-related costs while clinical outcomes might not be considered clinically significant.
